# Long COVID-19 Syndrome Severity According to Sex, Time from the Onset of the Disease, and Exercise Capacity—The Results of a Cross-Sectional Study

**DOI:** 10.3390/life13020508

**Published:** 2023-02-11

**Authors:** Elżbieta Paradowska-Nowakowska, Danuta Łoboda, Krzysztof S. Gołba, Beata Sarecka-Hujar

**Affiliations:** 1 Department of Cardiac Rehabilitation, “Ustron” Health Resort, 43-450 Ustron, Poland; 2 Department of Electrocardiology and Heart Failure, Medical University of Silesia, 40-635 Katowice, Poland; 3 Department of Electrocardiology, Upper-Silesian Medical Centre, 40-653 Katowice, Poland; 4 Department of Basic Biomedical Science, Faculty of Pharmaceutical Sciences in Sosnowiec, Medical University of Silesia, 41-200 Sosnowiec, Poland

**Keywords:** post-COVID-19 syndrome, long COVID-19 syndrome, symptoms

## Abstract

Symptoms of long COVID-19 syndrome (long COVID-19) are reported by 80% of convalescents up to several months after contracting the coronavirus-19 disease (COVID-19). The study aimed to assess the frequency and correlations of long COVID symptoms with sex, disease severity, time since the onset of the disease, and exercise capacity in a population of Polish convalescents hospitalized as a part of a rehabilitation program after COVID-19. The retrospective analysis was carried out based on medical records concerning reported symptoms, comorbidities, exercise capacity, fatigue and dyspnea on Borg’s scale, arterial oxygen saturation (SpO_2_), spirometric parameters, chest X-rays/computed tomography scans, systolic pulmonary artery pressure, and left ventricular ejection fraction. The study involved 471 patients aged 63.83 ± 9.93 years who had been hospitalized 191.32 ± 75.69 days from the onset of COVID-19, of which 269 (57.1%) were women. The most common symptoms were fatigue (99.57%), dyspnea (99.36%), and myalgia (97.03%). Women reported more symptoms than men (*p* < 0.001) and rated their fatigue as more severe (*p* = 0.021). Patients with depressed moods reported more physical symptoms than others (*p* < 0.001). Most long COVID symptoms, including dyspnea, fatigue, and depressive symptoms, were found with the same frequency in patients 12–24 weeks and >24 weeks after recovery (*p* = 0.874, *p* = 0.400, and *p* = 0.320, respectively), regardless of acute COVID-19 severity (*p* = 0.240, *p* = 0.826, and *p* = 0.108, respectively). Dyspnea severity correlated with forced vital capacity (FVC) (r = −0.153, *p* = 0.005), and forced expiratory volume in one second (FEV1) (r = −0.142, *p* = 0.008). Fatigue severity correlated with impaired FVC and FEV1 (both r = −0.162, *p* = 0.003). Fatigue and dyspnea inversely correlated with the distance in a six-minute walk test (r = −0.497, *p* < 0.001, and r = −0.327, *p* < 0.001). In conclusion, in our cohort, long COVID symptoms are more common in women. Dyspnea/fatigue and depressive symptoms do not tend to subside after an average six-month recovery period. The intensity of perceived fatigue may be exaggerated by the coexistence of neuropsychiatric disorders. Increased fatigue and dyspnea correlate with impaired spirometric parameters and significantly affects convalescents’ exercise capacity.

## 1. Introduction

As many as 80% of those who recover from coronavirus 2019 disease (COVID-19) experience ailments related to the disease that persist for up to several months [1,2,3,4]. These ailments include worsening exercise capacity, muscle pain, dyspnea, chronic cough, chest pain, heart palpitations, increased/unstable blood pressure, anosmia, ageusia, and alopecia [1,2,4]. However, some of the most common symptoms reported by convalescents are chronic fatigue as well as mood and cognitive and mental disturbances; these symptoms negatively affect the quality of life and problematize the return to everyday duties [3,5,6,7,8,9,10,11]. All these symptoms are referred to as long COVID-19 syndrome (long COVID-19) if they persist or develop de novo at least 12 weeks after the initial severe acute respiratory syndrome coronavirus-2 (SARS-CoV-2) infection and last for at least two months without other explanation [12]. Long COVID-19 affects convalescents from COVID-19, regardless of the severity of the acute illness [13,14]. Female sex, multiple early symptoms, lower SARS-CoV-2 IgG titer, biomarkers (e.g., D-dimer, CRP, and lymphocyte count), and previous psychiatric disorders were listed as risk factors [14,15].

The pathogenesis of long COVID-19 has not yet been resolved. Some authors associate it with immune dysregulation, an autoimmune process, viral persistence, long-term tissue damage, and thrombotic/post-thrombotic changes that persist after the acute infection phase [16,17,18,19]. The neurotropic and neuroinvasive properties of SARS-CoV-2 can explain some neurological symptoms of long COVID (e.g., the loss of smell and taste, as well as lacrimal, salivary, and auditory dysfunctions [20,21,22]). SARS-CoV-2 can infect neurons and neuroglia through the nasal and oral epithelium and retrogradely enter the central nervous system via cranial nerves [20,23]. The systemic inflammatory response to COVID-19 is considered another factor leading to central and peripheral nervous system damage and determining the persistence of neurological and cognitive symptoms [21,24,25]. More recent studies examine the relationship of long COVID-19 with critical illness neuropathy/critical illness myopathy/post-intensive care syndrome (PICS) [21,26] or muscle deconditioning on the recovery of an acute inflammatory disease and prolonged bed rest [27,28,29,30]. The contributions of encephalomyelitis/chronic fatigue syndrome (ME/CFS) [3,31,32,33], post-traumatic syndrome (PTSD) [34,35,36], and depression and anxiety disorders [8,9,10,11] are also considered due to some discrepancies between the severity of subjective symptoms and objective measures of lung and heart functions.

The present study aimed to assess the frequency and correlations of long COVID-19 symptoms with gender, disease severity, time since the onset of the disease, and exercise capacity in the population of Polish convalescents hospitalized as part of a comprehensive cardiopulmonary rehabilitation (CR) program after COVID-19.

## 2. Materials and Methods

### 2.1. Study Design and Setting

The study was cross-sectional and retrospective. The medical records of patients participating in the CR program at the Cardiac Rehabilitation Department of the “Ustroń” Health Resort from July 2021 to April 2022 were searched. The CR was completed as part of the National Health Fund (NHF) program intended for patients after symptomatic COVID-19, up to 12 months from the diagnosis. The recruitment process was conducted by an experienced pulmonologist (E.P.-N.).

The study was approved by the Bioethics Committee of the Medical University of Silesia in Katowice (PCN/CBN/0022/KB1/68/21 and PCN/CBN/0052/KB1/68/I/21/22).

### 2.2. Patients

Patients (Caucasians aged ≥ 18 years) were admitted to the Health Resort from home, based on a referral from a general practitioner and a scheduled basis. Eligibility criteria for the hospitalization and CR were fully consistent with the recommendations of the NHF [37]. These were as follows: (1) complications or consequences of symptomatic SARS-CoV-2 infection in the respiratory system, cardiovascular system, nervous system, or musculoskeletal system, or (2) decrease in muscle strength assessed using the Medical Research Council scale, or (3) persistent dyspnea of the intensity of 2–3 on the modified Medical Research Council dyspnea scale.

In all patients, active COVID-19 was excluded based on qualitative tests for the presence of SARS-CoV-2 antigen in nasopharyngeal swabs [38]. The exclusion criterion from the study group was the lack of or incomplete medical documentation concerning the course of COVID-19 or failure to meet the WHO long-COVID criteria [12].

Each patient gave written informed consent to participate in the study.

### 2.3. Analyzed Variables, Source of Data, and Measurement

In the present study, the following variables were analyzed:

(1) symptoms of the long COVID-19 syndrome (i.e., data were gathered on the presence or absence of the following long COVID-19 symptoms reported/felt/treated by patients: fatigue/weakness, dyspnea, cough, myalgia, chest pain, palpitations, increased/unstable blood pressure, concentration disorders/memory deterioration, depressed mood/sleep disturbances, loss/impairment of smell, hair loss, deterioration of eyesight and hearing, and dizziness.);
(2) data on comorbidities (i.e., cardiovascular, pulmonary, liver, renal, and neurological diseases, and diabetes (DM);
(3) continuous variables (age, BMI, exercise capacity, baseline spirometric parameters).

Information on long COVID-19 symptoms as well as comorbidities was obtained through questionnaires.

Hypertension was defined as systolic blood pressure ≥ 140 mmHg or diastolic blood pressure ≥ 90 mmHg, or the use of antihypertensive drugs [39]. The exercise capacity was assessed as the distance in the six-minute walk test (6MWT). This capacity was presented as a percentage of the predicted value for age and sex, calculated according to Enright’s formula [40]. Exercise tolerance during the 6MWT was presented and rated according to the Borg Rating of Perceived Exertion scale (Borg scale) with the assessment of two components (fatigue and dyspnea) [41]. Arterial oxygen saturation (SpO_2_) was measured by pulse oximetry at rest and after the 6MWT. Data on left ventricular ejection fraction (LVEF), as assessed by Simpson’s biplane disc summation method [42], were included. Data on the systolic pulmonary artery pressure and the risk of pulmonary hypertension, assessed based on the continuous wave Doppler tricuspid regurgitation systolic jet velocity, inferior vena cava size and collapsibility, pulmonary velocity acceleration time, and right atrium surface area [43], were taken into account. The following baseline spirometric parameters [44] were considered: (1) forced vital capacity compared to the predicted value for age and sex (FVC% pred), (2) forced expiratory volume in one second compared to the predicted value for age and sex (FEV1% pred), and (3) the FEV1/FVC ratio.

In addition, chest X-rays and lung computed tomography (CT) data were reviewed. These included the percentage of lung area involved during acute COVID-19 and the presence of parenchymal/fibrotic changes on follow-up (if available).

### 2.4. Criteria for Dividing COVID-19 Convalescents into Subgroups

Patients were analyzed in subgroups according to:

(1) sex (men, women);
(2) severity of acute COVID-19 (Stage I, Stage II, Stage III, and Stage IV);
(3) time from the disease onset (12–24 weeks, >24 weeks).

The four levels of severity (grades I–IV) of acute COVID-19 were established according to the Polish Society of Epidemiologists and Infectiologists guidelines [38]. Patients with Stage I of the disease were asymptomatic or mildly symptomatic (mild COVID-19), with a SpO_2_ of ≥94% on room air. Patients with Stage II (moderate COVID-19) developed a flu-like clinical syndrome, with or without mild atypical pneumonia, and had a SpO_2_ of 90–94% on room air. Patients with Stage III (severe COVID-19) required hospitalization for hypoxic respiratory failure, with an SpO_2_ of <90% on room air and/or ≥50% lung infiltration on CT scan and/or pulmonary embolism. Patients with Stage IV (critical COVID-19) required treatment in the intensive care unit due to presenting acute respiratory distress syndrome and/or septic shock and/or multi-organ failure.

Patients were also divided into two subgroups according to time after the acute phase of COVID-19, i.e., 12–24 weeks and >24 weeks from disease onset.

### 2.5. Statistical Analysis

Statistical analyses were performed using Statistica 13 (StatSoft; Statistica, Tulsa, OK, USA). The continuous variables were expressed as mean (M) and standard deviation (SD), while categorical variables were shown as absolute numbers (n) and percentages (%). The normality of the distribution of the variables was assessed using the Shapiro–Wilk W test. Depending on the normality distribution or its lack, the comparisons of continuous variables (i.e., age, BMI, exercise capacity, and baseline spirometric parameters) between the two groups were made using the Student *t*-test or the Mann–Whitney U test, respectively. To compare continuous variables between more than two groups of patients, ANOVA or the Kruskal–Wallis H tests were used, depending on the variable’s distribution. In the case of significant differences observed using ANOVA, the post-hoc Scheffe’s test was used. In turn, when a statistical significance was observed in the Kruskal–Wallis test, the Mann–Whitney U test was used for post-hoc pairwise comparison. The stochastic independence χ^2^ test was used to compare categorical variables (i.e., frequencies of symptoms of post-COVID-19 syndrome and comorbidities) between the study groups. To establish possible correlations between continuous variables, Spearman’s correlation coefficients were estimated. The significance level was set at *p* ≤ 0.05 in all statistical tests.

We performed a power analysis during the study, on the basis of differences in the selected parameters, i.e., palpitations, increased/unstable blood pressure, depressed mood/sleep disturbance, hair loss, and dizziness between men and women in the total group using a two-tailed test with a significance level of 0.05. Depending on the parameter, we obtained different values of power within a range from 65% to 80%. Thus, we established the power of the study as sufficient.

## 3. Results

### 3.1. Characteristics of the Study Group

We assessed the medical records of 558 consecutive patients who qualified for rehabilitation (Caucasians aged ≥ 18 years). Of these, 87 patients not meeting the criteria for long-COVID-19 or with missing/incomplete medical documentation regarding the course of COVID-19 were excluded. Ultimately, the study group consisted of 471 patients with a mean age of 63.83 years ± 9.93. Of these, 269 (57.1%) were women. Patients participated in the rehabilitation program at a mean time of 191.32 days ± 75.69 from the onset of COVID-19. Table 1 demonstrates the characteristics of the total group of COVID-19 convalescents and sex subgroups.

The male and female participants were of similar age and BMI. The capacity to exercise was reduced below the lower limit of normal in 159 participants (33.76%). In turn, LVEF < 50% was recorded in only 11 (5.07%) patients. Abnormal spirometry results were found in 71 (21.13%) patients for FVC (i.e., <80% pred), 110 (32.74%) patients for FEV1 (i.e., <80% pred), and one patient (0.3%) for FEV1/FVC ratio (i.e., <70%). LVEF and exercise capacity were higher in women than in men. Additionally, women had better spirometric and chest X-rays/CT results than men. No significant differences in remained parameters mentioned in Table 1 were observed between men and women.

Table 2 demonstrates the patients’ comorbidities. A greater percentage of women suffered from hypertension before COVID-19 than did men.

### 3.2. Distribution of Symptoms of the Long COVID-19 Syndrome in the Total Study Group

**Figure 1:**
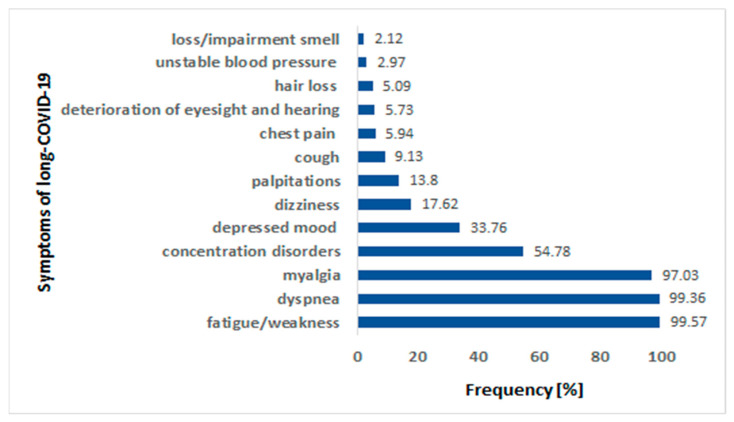
The frequencies of analyzed symptoms of long COVID-19 syndrome in the study cohort. COVID-19—coronavirus disease 2019.

The patients suffered from 2 to 10 symptoms after COVID-19 (4.5 on average). The most commonly observed symptom of long COVID-19 was fatigue or weakness, reported by almost all patients (469 out of 471, i.e., 99.57%). A similar percentage of patients (i.e., 99.36%) reported dyspnea and myalgia. The less frequent symptoms of long COVID-19 in our study group were as follows: chest pain, deterioration of eyesight and hearing, hair loss, unstable blood pressure, and loss/impaired smell. Figure 1 shows the frequencies of all the analyzed symptoms of long COVID-19 syndrome.

The severity of fatigue on the Borg’s scale correlated positively with age (r = 0.135, *p* = 0.003), while the severity of dyspnea did not (*p* = 0.372). Neither severity of fatigue nor dyspnea severity was related to LVEF (*p* = 0.852 and *p* = 0.308, respectively). Patients with depressed mood/sleep disorders reported more symptoms than patients without depressed mood/sleep disorders on average (5.43 ± 0.88 vs. 3.98 ± 1.01, *p* < 0.001), including, more often, concentration disorders (*p* < 0.001) and dizziness (*p* = 0.044). However, the severity of fatigue/dyspnea assessed on the Borg scale was comparable in this group than in the others, i.e., fatigue 4.79 ± 2.13 vs. 4.62 ± 2.06, *p* = 0.305; dyspnea 3.45 ± 2.53 vs. 3.39 ± 2.63, *p* = 0.845.

### 3.3. Distribution of Symptoms of the Long COVID-19 Syndrome According to Sex

Table 3 demonstrates the frequencies of the reported symptoms of long COVID-19 in sex subgroups. We observed that myalgia, palpitations, increased/unstable blood pressure, concentration disorders/memory deterioration, depressed mood/sleep disturbances, hair loss, and dizziness were significantly more common in women than in men. The remaining symptoms did not differ between the sexes. Women reported from 2 to 10 symptoms (4.8 on average) while men reported from 2 to 7 symptoms (4.1 on average) (*p* < 0.001). In addition, women rated their fatigue as being more severe on the Borg scale than men.

### 3.4. Distribution of Symptoms of the Long COVID-19 Syndrome According to Disease Severity

The prevalence of most symptoms of long COVID-19 did not differ between the groups depending on the severity of the acute phase of COVID-19 (Table 4).

Only the presence of cough and loss/impaired smell after COVID-19 significantly differentiated the patients regarding the disease severity. Cough was the most common in patients with Stage I of the disease (10.26%) and less common in patients with Stage III of the disease (1.49%; *p* = 0.036). The greatest number of patients with Stage I of the disease (3.85%) suffered from loss/impaired smell, while none of patients with Stage III and IV of the disease (*p* = 0.037) had loss/impaired smell.

The number of reported symptoms and dyspnea/fatigue severity did not differ between inpatients or outpatients during the acute COVID-19 period (*p* = 0.184, *p* = 0.623, and *p* = 0.370, respectively). Additionally, the mean number of symptoms and dyspnea/fatigue severity did not correlate with the number of hospital days (*p* = 0.801, *p* = 0.559, and *p* = 0.746, respectively). The percentage of lung area with parenchymal abnormalities confirmed by chest X-rays/CT scans in acute COVID-19 or the presence of parenchymal changes/interstitial fibrosis on follow-up chest X-ray did not affect dyspnea (*p* = 0.463 and *p* = 0.877, respectively) and fatigue (*p* = 0.952 and *p* = 0.549, respectively) severity during the recovery. Dyspnea severity did not correlate with SpO_2_ values at rest and post-exercise. On the contrary, dyspnea severity was inversely related to FVC% pred (r = −0.153, *p* = 0.005) and FEV1% pred (r = −0.142, *p* = 0.008). Similar correlations were observed between fatigue severity and FVC% pred (r = −0.162, *p* = 0.003) and FEV1% pred (r = −0.162, *p* = 0.003). Fatigue and dyspnea severity inversely correlated with the distance at the 6MWT (r = −0.497, *p* < 0.001, and r = −0.327, *p* < 0.001, respectively).

### 3.5. Distribution of Symptoms of Long COVID-19 Syndrome According to Time from Disease Onset

The prevalence of the symptoms of long COVID-19 syndrome according to the time from the acute phase of the disease is shown in Table 5.

Cough and concentration disorders/memory deterioration were the only two symptoms that differentiated subgroups of patients depending on the time from the disease’s acute phase (i.e., 12–24 weeks and >24 weeks). Cough was most common in patients >24 weeks after acute COVID-19 and less in patients 12–24 weeks after (11.97% vs. 5.64%, respectively, *p* = 0.036). In turn, concentration disorders/memory deterioration were the most common in patients 12–24 weeks (66.15%) and less common in patients being >24 weeks (48.29%) (*p* < 0.001).

## 4. Discussion

Our work presents an analysis of the cohort of 471 convalescents in which the proportions of mild (49.68%), moderate (32.06%), severe (14.23%), and critical (3.82%) COVID-19 cases corresponded to the typical distribution of severity of acute disease phases in the general population [1,3]. The results of this study indicate that symptoms of long COVID-19 are more common in women. Women are more prone to concentration disorders/memory deterioration, depressed mood/sleep disturbances, myalgia, elevated/unstable blood pressure, palpitations, dizziness, and hair loss. The prevalence of mood/cognitive disorders is high among post-COVID-19 convalescents. Patients with depressed mood/sleep disorders report more symptoms than others, including impaired concentration and dizziness. Chronic fatigue and dyspnea do not correlate with the severity of acute COVID-19 and do not tend to subside after an average six-month recovery period. Increased dyspnea and fatigue inversely correlate with FVC% pred and FEV1% pred but not with LVEF. Chronic fatigue/dyspnea significantly affects the exercise capacity of convalescents measured as a distance in 6MWT.

Dyspnea/fatigue is the most frequently reported problem that significantly limits the capacity to exercise in COVID-19 convalescents [1,2,3,4,5]. However, the severity of dyspnea/fatigue does not appear to be related to either the severity of acute COVID-19 or the extent of the dysfunction of the heart and lungs during the recovery period [13,14,27,45]. Dyspnea/fatigue is often accompanied by symptoms of depression and anxiety and cognitive disorders [5,6,7,8,10,11,21]. This points to the complex etiopathogenesis of long COVID.

On the one hand, we discuss patients with a history of acute interstitial pneumonia, often affecting either a large area of the lung parenchyma or complicated by ARDS [46,47]. Residual lung parenchymal abnormalities on CT scans in up to 91% of patients are known to be present three months after discharge from the hospital [48,49,50]. These abnormalities included residual ground-glass opacities in 86%, bronchiectasis in 60%, lines or bands in 64%, and radiological signs of fibrosis in 26% of participants. The presence and extent of these changes correlate with a decreased diffusion capacity of the lungs for carbon monoxide (DLCO) [48]. DLCO is a commonly reported problem in long COVID-19 patients, with the decrease in DLCO being proportional to the severity of the acute phase of the disease [1,49,50,51,52] and persisting for one year in 35% of patients [53]. Some authors also describe mildly decreased FVC and FEV1 [52,54]. Furthermore, COVID-19-associated coagulopathy is responsible for thrombotic complications, including pulmonary embolism, in 20–30% of patients with acute COVID-19 [18,19,55,56]. The increased risk of new thrombotic complications persists for up to 110 days in convalescents [19]. These factors can affect lung function during recovery.

Lung parenchyma abnormalities/interstitial fibrosis were seen on the control chest X-rays/CT scans of 64.9% of patients in our study. Dyspnea was found with the same frequency in patients >24 weeks from the onset of COVID-19 relative to patients 12–24 weeks post-infection. This characteristic may suggest post-thrombotic changes, the persistence of parenchymal abnormalities, or the development of late pulmonary complications such as pulmonary fibrosis. However, RVSP assessed by echocardiography was correct, and none of the patients had a high risk of pulmonary hypertension. A total of 33.8% of patients had a significantly reduced capacity to exercise. The mean distance in 6MWT assessed as 373.7 m was lower than in a comparable age group of patients we evaluated during rehabilitation after myocardial infarction (627.0 m) [57]. Mean spirometric parameters in the study group were mildly reduced (FVC% 93.2% pred and FEV1% 88.6% pred), although they were lower than 80% of the predicted value in 21.1% (FVC) and 32.7% (FEV1) of participants. The decreased FVC% pred and FEV1% pred were principal parameters affecting patients’ capacity and tolerance for exercise.

It is noteworthy that changes in the lung parenchyma/thrombotic changes/restrictive pulmonary disease may not be the only causes of dyspnea/fatigue in long COVID-19 patients. There is some evidence of impaired muscle strength in convalescents after COVID-19 [1,58,59,60]. Especially after severe and critical COVID-19, impaired muscle strength and sarcopenia may be due to immune-mediated focal myofiber necrosis or atrophy proportional to viral load, and impaired metabolic function of skeletal muscles [58,59,60], malnutrition [59], and the effects of mechanical ventilation, sedative drugs, and neuromuscular blockade in PICS [16,26,59]. Muscle weakness associated with COVID-19 can also be caused by deconditioning on the recovery of an acute inflammatory disease and prolonged bed rest. The concept of deconditioning as a significant cause of unexplained dyspnea in COVID-19 convalescents has been documented based on a cardiopulmonary exercise test (CPET) [28,29,30]. Naeije et al. [27] summarized the results of several studies using CPET (Rinaldo et al. [28], Skjørten et al. [29], Motiejunaite J et al. [30], and others, over 500 patients in total), showing the profile of respiratory disorders typical of muscle deconditioning after an acute illness. It includes mean FEV1 of 97% of pred, DLCO of 83% of pred, peak oxygen consumption of 82% of pred, an anaerobic threshold of 50% of peak oxygen consumption, a slope of minute ventilation to CO_2_ production of 30, preserved respiratory reserve, and moderately reduced maximum heart rate. In addition, Mancini et al. [33] described impaired circulatory response to exercise (possible in the course of the thromboembolic pulmonary disease), abnormal ventilatory pattern, resting hypocapnia in patients with long COVID-19 and normal pulmonary function tests, chest X-rays/CT scans, and LVEF. Muscle weakness and muscle deconditioning may affect spirometric parameters, perceived dyspnea, exercise capacity, and fatigue severity [27,58].

In addition, Ortelli et al. [24] demonstrated that interleukine-6-related hyper-inflammation might play a role in central neuromotor and cognitive fatigue, apathy, and executive/motivation dysfunction in long COVID by downregulating gamma-aminobutyric acid (GABA) receptors. Mental fatigue may also increase the perception of exertion and impair performance during endurance exercises, regardless of the peripheral nervous system disorders. The systemic inflammatory response to COVID-19, the autoimmune response, and non-depolarizing neuromuscular blocking agents are also the cause of peripheral sensory and motor polyneuropathy, Guillain-Barre syndrome, and critical illness neuropathy/myopathy that impair cardiorespiratory fitness and exercise tolerance [21,25,58].

Cardiovascular manifestations of acute COVID-19 are reported in 20–30% of hospitalized patients, including myocarditis with decreased LVEF or late gadolinium enhancement on magnetic resonance [61]. It is known that late CV abnormalities such as left and right ventricular systolic and diastolic dysfunction, pulmonary hypertension, or pericardial effusions can be correlated with the severity of acute COVID-19, the time since the onset of the disease, and the number of symptoms of long COVID-19 [62,63]. They are caused by chronic post-infectious perimyocarditis, arterial wall inflammation with endothelial dysfunction, and microthrombosis [62]. In our study, no correlation was observed between dyspnea and fatigue severity or 6MWT distance and LVEF, probably because few patients experienced systolic heart failure after COVID-19. Similarly, in the study by Baum et al., the fatigue severity did not correlate with LVEF and left ventricular global longitudinal strain or with cardiac biomarkers, i.e., high-sensitivity troponin T and n-terminal pro-brain natriuretic peptide [45].

Conversely, the COVID-19 pandemic, due to its scope, lethal complications, initially poorly understood etiological factors, lack of causal treatment, concerns about the effectiveness and safety of vaccinations, and social isolation during the quarantine and lockdown period, generated an unprecedented level of distress and anxiety [10,11,21].

The frequency of depressive symptoms 12 weeks after recovery from COVID-19 is high and ranges from 11% to 28% [7]. It is also known that patients with preexisting depression or anxiety are more likely to suffer from chronic fatigue [8,13] and report more physical symptoms of long COVID-19 [8]. Many authors report a higher frequency of such symptoms in women [2,5,11]. In our analysis, women reported more symptoms than men, including more severe fatigue, even though men have a worse capacity for exercise, worse spirometric tests, and a more severe course of the acute phase of COVID-19. Women also reported depressive symptoms more often than men.

Mood disorders did not tend to subside over time. Male and female patients >24 weeks from the disease showed the same frequency of depressed mood/sleep disturbances as patients between 12 and 24 weeks of the disease. Persistent, chronic ailments, and poor exercise tolerance, which make it hard to return to everyday duties, may have a negative impact on mood, so the prevalence of neuropsychiatric symptoms may increase over time [6,31]. Jason et al. [31] and Davis et al. [3] reported that fatigue and several neurocognitive symptoms might persist and even worsen over time in susceptible individuals, similar to encephalomyelitis/chronic fatigue syndrome (ME/CFS). Davis et al. [3] reported post-exercise physical or mental malaise (ME/CSF symptoms) in 89.1% of COVID-19 survivors. In most respondents, the disability lasted a few days, but in as many as 15%, it prolonged to several weeks. Mancini et al. [33] described full symptoms of ME/CFS in 46% of patients with long COVID-19, on average 8.9 (SD 3.3) months after acute infection. So far, ME/CSF, although not related to COVID-19, has also been observed more frequently in women [32]. The leading symptom of ME/CSF is fatigue with a significant impairment of the ability to engage in educational, professional, personal, or social activities at a pre-disease level that persists for more than six months. One reported cause of this syndrome is stress or immune activation after an infectious disease, including infection with common viruses, e.g., Epstein–Barr virus, cytomegalovirus, enterovirus, human herpesvirus-6, human parvovirus B19, and hepatitis C virus [32,64]. Similar symptoms have also been observed by Lam et al. [65] and Moldovsky et al. [66] in severe acute respiratory syndrome (SARS, 2005), where 27% of survivors met the criteria for ME/CFS. It cannot be ruled out that, as Ortelli et al. [24] described, reduced activity of intracortical GABAergic circuits related to neuroinflammation could underlie neuromotor fatigue and the abnormal fatigue perception in the ME/CSF.

Another problem that may exacerbate long COVID-19 symptoms is PTSD [67]. PTSD can develop after exposure to traumatic events perceived as life-threatening and is characterized by, among other things, persistent re-experiencing of the event, causing considerable distress and functional impairment. PTSD occurs especially among patients hospitalized due to severe COVID-19, including those hospitalized in the intensive care unit [34,35]. Harenwall et al. [36] confirm that PTSD significantly interacts with dyspnea to predict fatigue severity in long COVID-19. In the presented study, patients after a severe and critical course of the acute phase of COVID-19 (Stages III and IV) did not report symptoms of long COVID-19 more often than patients with a mild course of infection. Townsend et al. [13] and Mandal et al. [14] also stated that symptoms of long COVID-19, including chronic fatigue, can occur regardless of the severity of acute COVID-19. PTSD is also reported in the general population (not diagnosed with COVID-19) due to the pandemic’s psychosocial impact [10].

The overlap of ME/CFS, PTSD, and depression and anxiety disorders may explain some discrepancies between the severity of subjective symptoms, including dyspnea/fatigue, the severity of acute COVID-19, and objective measures of lung and heart function during recovery. They may also explain the higher incidence of long COVID-19 symptoms in women.

Other symptoms of long COVID-19, such as muscle pain, chest pain, palpitations, unstable blood pressure, and dizziness, were also found in our study with a similar frequency in the surveyed patients at 12–24 weeks, and >24 weeks after acute COVID-19. However, concentration disorders/memory deterioration were less common in patients >24 weeks after the disease. Davis et al.’s analysis [3] documented that the likelihood of “severe” and “very severe” symptoms is higher during the acute phase of the infection and decreases; however, symptoms of long COVID-19 rated by patients as “moderate” and “mild” may increase later. Furthermore, the probability of their persistence for more than 35 weeks is 91.8%, regardless of sex.

The overlapping of somatic and psychiatric symptoms in the course of long COVID-19 requires multidirectional rehabilitation [68,69,70]. The CR programs bring improvements in cardiorespiratory fitness, musculoskeletal fitness, and exercise tolerance, regardless of the time from the onset of acute COVID-19 symptoms to the CR, also in the long COVID-19 population [71,72,73,74,75]. Resistance training is an effective treatment for sarcopenia, improving muscle mass, quality, and strength in long COVID-19 patients [76,77]. Regular exercise helps improve mood, reduces psychological stress, and modulates pain perception, thereby improving the quality of life [75,78,79]. In addition, CR offers a chance for a timely diagnosis and the treatment of depression, anxiety, and PTSD, as well as breaking social isolation [69]. The CR also contains elements of dietary and pro-health counseling on comorbidities [68,69].

Our study had some limitations. First, our retrospective analysis was based on survey and medical record data; this hindered the free selection of diagnostic methods, potentially impacting the reliability of symptom prevalence estimation. The medical records did not contain questionnaires on the severity of depressive and neurocognitive disorders. Second, the inclusion criteria for the study were in line with the eligibility criteria for the post-COVID-19 CR program of the NHF. Based on these criteria, the proportion of patients reporting exertional dyspnea, fatigue, and muscle weakness may be slightly higher in the study group than in the general population. In addition, a trend towards a higher percentage of patients with cardiovascular and metabolic diseases in the convalescent group is possible, as these comorbidities favor the development of COVID-19 complications [80,81]. It is also worth noting that the Polish population has been classified as a high-risk group for atherosclerotic cardiovascular diseases [82], and the prevalence of these comorbidities may be higher than in the Western European population. However, the presented group participating in CR appears to be a representative sample of the Polish COVID-19 convalescents, considering the typical distribution of COVID-19 severity and non-restrictive admission criteria to the Health Resort. The above bias does not seem to affect the correlations between long COVID-19 symptoms, including dyspnea and fatigue and sex, disease severity, the time elapsed since its onset, cardiac and lung capacity, or exercise capacity. Thirdly, in the study population, assessing lung parenchymal abnormalities or interstitial fibrosis was mainly limited to follow-up chest X-rays without high-resolution CT evaluation or lung capacity diffusion for carbon monoxide. Diagnostics based only on chest X-rays could have influenced the overdiagnosis of parenchymal/fibrotic changes in the study. Muscle strength was not assessed in the study. The assessment of exercise capacity in the 6MWT was used instead of the cardiopulmonary exercise test (CPET). The medium-term evaluation considered the average time from the onset of COVID-19 to the assessment, usually after six months. Long-term data require a separate study.

## 5. Conclusions

The present study, conducted in a group of Polish convalescents rehabilitated after COVID-19, indicates that long COVID-19 symptoms are more common in women. Chronic fatigue and dyspnea do not correlate with the severity of the acute phase of COVID-19 and do not tend to subside after an average six-month recovery period. Increased fatigue and dyspnea correlate with impaired spirometric parameters but not with left ventricular systolic function. The intensity of perceived fatigue and dyspnea seems disproportionately high considering the dysfunction of the heart and lungs, which may be related to the coexistence of neuropsychiatric disorders. Chronic fatigue and dyspnea significantly affect convalescents’ exercise capacity after COVID-19.

## Figures and Tables

**Table 1:** General characteristics of the total group of COVID-19 convalescents and sex subgroups.

	Total Group *N* = 471	Men *N* = 202 (42.89%)	Women *N* = 269 (57.11%)	*p*
Age (years), M ± SD	63.83 ± 9.93	64.21 ± 9.52	63.55 ± 10.23	0.487
BMI at admission (kg/m^2^), M ± SD	29.69 ± 4.85	29.32 ± 4.24	29.97 ± 5.25	0.174
6MWD (m), M ± SD	373.67 ± 84.24	389.13 ± 77.41	361.97 ± 87.41	**<0.001**
6MWD % pred (%), M ± SD	76.29 ± 16.31	73.27 ± 14.24	78.57 ± 17.41	**<0.001**
SpO_2_ at rest (%), M ± SD	96.74 ± 1.24	96.73 ± 1.19	96.75 ± 1.27	0.521
SpO_2_ post-exercise (%), M ± SD	97.35 ± 0.83	97.30 ± 0.81	97.39 ± 0.84	0.137
FVC % pred (%), M ± SD	93.16 ± 18.63	88.29 ± 15.81	96.62 ± 19.72	**<0.001**
FEV1 % pred (%), M ± SD	88.61 ± 18.64	86.72 ± 17.59	89.95 ± 19.28	0.165
FEV1/FVC ratio (%), M ± SD	101.25 ± 12.08	102.34 ± 11.29	100.44 ± 10.52	**0.027**
LVEF (%), M ± SD	57.31 ± 6.03	56.11 ± 6.94	58.19 ± 5.11	**0.033**
Time from disease onset (days), M ± SD	191.32 ± 75.69	191.40 ± 77.03	191.26 ± 74.81	0.799
Duration of rehabilitation (days), M ± SD	25.22 ± 7.66	25.18 ± 7.70	25.25 ± 7.65	0.991
Number of symptoms of long COVID-19 syndrome reported by patients, n (%)	4.47 ± 1.19	4.09 ± 1.05	4.74 ± 1.24	**<0.001**
Stage of the disease, n (%)				0.079
I	234 (49.68)	89 (44.06)	145 (53.90)	
II	151 (32.06)	67 (33.17)	84 (31.27)	
III	67 (14.23)	36 (17.82)	31 (11.52)	
IV	18 (3.82)	10 (4.95)	8 (2.97)	
Ranges of time from disease onset, n (%)				0.845
12–24 weeks	195 (4545)	86 (46.24)	109 (44.86)	
>24 weeks	234 (54.55)	100 (53.76)	134 (55.14)	
Percentage of lung area involved in CT in acute COVID-19 (%)	50.63 ± 22.26	55.44 ± 21.58	45.19 ± 22.90	**0.025**
Parenchymal changes on control chest X-ray/CT scans, n (%)	300 (63.69)	143 (71.86)	157 (59.92)	**0.010**

M—mean; SD—standard deviation; BMI—body mass index; COVID-19—coronavirus disease 2019; FVC—forced vital capacity; FEV1—forced expiratory volume in one second; % pred—the predicted value for age and sex; LVEF—left ventricular ejection fraction; 6MWD—the distance in the six-minute walk test; 6MWD % pred–the percentage of predicted distance in the six-minute walk test according to Enright et al. [40]; SpO2—arterial oxygen saturation in pulse oximetry measurements; CT—computed tomography. Significant differences are in bold.

**Table 2:** Underlying medical conditions of the patients.

	Total Group *N* = 471	Men *N* = 202	Women *N* = 269	*p*
Coronary artery disease, n (%)	60 (12.74)	25 (12.38)	35 (13.01)	0.885
Myocardial infarction, n (%)	18 (3.82)	9 (4.45)	9 (3.35)	0.623
Hypertension, n (%)	239 (50.74)	91 (45.05)	148 (55.01)	**0.038**
Atrial fibrillation, n (%)	31 (6.58)	14 (6.93)	17 (6.32)	0.823
Ischemic stroke, n (%)	11 (2.33)	2 (0.99)	9 (3.35)	0.195
Asthma/Chronic obstructive pulmonary disease, n (%)	38 (8.07)	15 (7.43)	23 (8.55)	0.907
Diabetes mellitus, n (%)	83 (17.62)	31 (15.35)	52 (19.33)	0.426
Gout/hyperuricemia, n (%)	30 (6.37)	10 (4.95)	20 (7.43)	0.388
Pulmonary embolism, n (%)	9 (1.91)	3 (1.48)	6 (2.23)	0.867
Venous thrombosis, n (%)	7 (1.49)	3 (1.48)	4 (1.49)	0.747

Significant differences are in bold.

**Table 3:** The frequencies of the reported symptoms of the long COVID-19 syndrome according to sex.

Symptoms of the Long COVID-19 Syndrome	Men *N* = 202	Women *N* = 269	*p*
Fatigue/weakness, n (%)	201 (99.50)	268 (99.63)	0.608
Fatigue, Borg’s scale (pts.), M ± SD	4.41 ± 2.06	4.88 ± 2.08	**0.021**
Dyspnea, n (%)	201 (99.50)	267 (99.26)	0.803
Dyspnea, Borg’s scale (pts.), M ± SD	3.29 ± 2.55	3.51 ± 2.62	0.377
Cough, n (%)	15 (7.43)	28 (10.41)	0.342
Myalgia, n (%)	192 (95.05)	265 (98.51)	0.055
Chest pain, n (%)	11 (5.44)	17 (6.32)	0.842
Palpitations, n (%)	15 (7.43)	50 (18.59)	**<0.001**
Increased/unstable blood pressure, n (%)	2 (0.99)	12 (4.46)	**0.030**
Concentration disorders/memory deterioration, n (%)	100 (49.50)	158 (58.74)	**0.049**
Depressed mood/sleep disturbance, n (%)	54 (26.73)	105 (39.03)	**0.007**
Loss/impaired smell, n (%)	2 (0.99)	8 (2.97)	0.248
Hair loss, n (%)	2 (0.99)	22 (8.18)	**<0.001**
Dizziness, n (%)	22 (10.89)	61 (22.68)	**0.001**
Deterioration of eyesight and hearing, n (%)	11 (5.44)	16 (5.95)	0.975

COVID-19—coronavirus disease 2019; Borg’s scale—Borg’s Rate of Perceived Exertion scale. Significant differences are in bold.

**Table 4:** The frequencies of the reported symptoms of the long COVID-19 syndrome according to the stage of the disease’s acute phase.

Symptoms of the Long COVID-19 Syndrome	Stage I *N* = 234	Stage II *N* = 151	Stage III *N* = 67	Stage IV *N* = 18	*p*
Fatigue/weakness, n (%)	233 (99.57)	150 (99.34)	67 (100.00)	18 (100.00)	0.826
Fatigue, Borg’s scale (pts.), M ± SD	4.72 ± 2.07	4.61 ± 2.06	4.78 ± 2.07	4.28 ± 2.56	0.816
Dyspnea, n (%)	231 (98.72)	151 (100.00)	67 (100.00)	18 (100.00)	0.240
Dyspnea, Borg’s scale (pts.), M ± SD	3.41 ± 2.50	3.54 ± 2.65	3.15 ± 2.70	3.29 ± 3.04	0.744
Cough, n (%)	24 (10.26)	15 (9.93)	1 (1.49)	3 (16.67)	**0.036**
Myalgia, n (%)	230 (98.29)	145 (96.03)	64 (95.52)	17 (94.44)	0.425
Chest pain, n (%)	10 (4.27)	11 (7.28)	5 (7.46)	2 (11.11)	0.441
Palpitations, n (%)	25 (10.68)	30 (19.87)	8 (11.94)	2 (11.11)	0.087
Increased/unstable blood pressure, n (%)	5 (2.14)	7 (4.64)	1 (1.49)	1 (5.56)	0.420
Concentration disorders/memory deterioration, n (%)	127 (54.27)	87 (57.62)	37 (55.22)	7 (38.89)	0.503
Depressed mood/sleep disturbance, n (%)	91 (38.89)	41 (27.15)	22 (32.84)	5 (27.78)	0.108
Loss/impaired smell, n (%)	9 (3.85)	1 (0.66)	0 (0.00)	0 (0.00)	**0.037**
Hair loss, n (%)	10 (4.29)	12 (8.00)	2 (2.99)	0 (0.00)	0.159
Dizziness, n (%)	50 (21.37)	24 (15.89)	8 (11.94)	1 (5.56)	0.092
Deterioration of eyesight and hearing, n (%)	15 (6.41)	9 (5.96)	3 (4.48)	0 (0.00)	0.468

COVID-19—coronavirus disease 2019; Borg’s scale—Borg’s Rate of Perceived Exertion scale. Significant differences are in bold.

**Table 5:** The frequency of the reported symptoms of long COVID-19 syndrome according to the time from the acute phase of the disease.

Symptoms of the Long COVID-19 Syndrome	12–24 Weeks *N* = 195	>24 Weeks *N* = 234	*p*
Fatigue/weakness, n (%)	193 (98.97)	234 (100.00)	0.400
Fatigue, Borg’s scale (pts.), M ± SD	4.73 ± 2.59	4.66 ± 2.01	0.924
Dyspnea, n (%)	193 (98.97)	233 (99.57)	0.874
Dyspnea, Borg’s scale (pts.), M ± SD	3.27 ± 2.59	3.51 ± 2.61	0.334
Cough, n (%)	11 (5.64)	28 (11.97)	**0.036**
Myalgia, n (%)	187 (95.90)	229 (97.86)	0.368
Chest pain, n (%)	9 (4.62)	16 (6.84)	0.440
Palpitations, n (%)	21 (10.77)	40 (17.09)	0.084
Increased/unstable blood pressure, n (%)	5 (2.56)	7 (2.99)	0.979
Concentration disorders/memory deterioration, n (%)	129 (66.15)	113 (48.29)	**<0.001**
Depressed mood/sleep disturbance, n (%)	61 (31.28)	85 (36.32)	0.320
Loss/impaired smell, n (%)	5 (2.56)	5 (2.14)	0.977
Hair loss, n (%)	11 (5.67)	9 (3.86)	0.516
Dizziness, n (%)	37 (18.97)	41 (17.52)	0.793
Deterioration of eyesight and hearing, n (%)	11 (5.64)	12 (5.13)	0.984

COVID-19—coronavirus disease 2019; Borg’s scale—Borg’s Rate of Perceived Exertion scale. Significant differences are in bold.

## Data Availability

The data presented in this study are available on request from the Department of Basic Biomedical Science, Faculty of Pharmaceutical Sciences in Sosnowiec, Medical University of Silesia in Katowice, (Poland). The data are not publicly available due to privacy restrictions.
